# Input graph: the hidden geometry in controlling complex networks

**DOI:** 10.1038/srep38209

**Published:** 2016-11-30

**Authors:** Xizhe Zhang, Tianyang Lv, Yuanyuan Pu

**Affiliations:** 1 School of Computer Science and Engineering, Northeastern University, Shenyang 110819, China; 2 College of Computer Science and Technology, Harbin Engineering University, Harbin 150001, China; 3 IT Center, National Audit Office, Beijing 100830, China

## Abstract

The ability to control a complex network towards a desired behavior relies on our understanding of the complex nature of these social and technological networks. The existence of numerous control schemes in a network promotes us to wonder: what is the underlying relationship of all possible input nodes? Here we introduce input graph, a simple geometry that reveals the complex relationship between all control schemes and input nodes. We prove that the node adjacent to an input node in the input graph will appear in another control scheme, and the connected nodes in input graph have the same type in control, which they are either all possible input nodes or not. Furthermore, we find that the giant components emerge in the input graphs of many real networks, which provides a clear topological explanation of bifurcation phenomenon emerging in dense networks and promotes us to design an efficient method to alter the node type in control. The findings provide an insight into control principles of complex networks and offer a general mechanism to design a suitable control scheme for different purposes.

Controlling complex networked systems is a fundamental challenge in natural, social sciences and engineered systems. A networked system is controllable if its state can be controlled from any initial state to a desired accessible state^1^,^2^ by inputting external signals from a few suitable selected nodes, which are called input nodes^3^,^4^,^5^,^6^. Existing works^3^ provide an efficient method based on maximum matching to find a **M**inimum **I**nput nodes **S**et (abbreviated *MIS*) used to fully control a network.

However, these works have primarily focused on analyzing single *MIS*^4^,^5^,^6^,^7^,^8^, while the underlying control relationships of nodes and *MIS*s remain elusive. Owing to the structural complexity of a network, its *MISs* are typically not unique and the number of *MIS*s are exponential to the size of the network^9^,^10^. The enumeration of all possible *MIS*s is a #P problem^11^ which requires high computational costs. A few works analyzed the node types in control^12^,^13^ and control capacities^10^ of input nodes. Moreover, although any of its *MIS*s are capable of fully controlling the network, they may composed of nodes with different topological properties, such as high-degree nodes^14^. The existence of physical constraints and limitations^15^ may also affect the choice of a suitable *MIS*. For example, when controlling an inter-bank market^16^,^17^, one may need certain specific input nodes to ensure that a *MIS* can be manipulated by a given organization; when controlling a protein interaction network^18^, some proteins cannot be used as input nodes because of technique limitation.

Given the existence of numerous *MIS*s in a network, a node can be classified based on its participation in *MIS*s^12^: 1. possible input node, which appear in at least one *MIS*; 2. redundant node, which never appear in any *MIS*. Previous works^12^ found that the dense networks exhibit a surprising bifurcation phenomenon, in which the majority of nodes are either redundant nodes or possible driver nodes. However, the origin of bifurcation phenomenon and the method of altering the type of nodes are still unknown.

Besides many approaches on controllability analysis of complex networks, the following questions are critical yet remain unknown: (*i*) what is the relationship between many available *MIS*s of a network? (*ii*) what topological structure determines whether a node is a possible input node? (*iii*) how to design suitable *MIS* with the desired nodes?

Here, we present input graph, a simple geometry but capable of revealing the complex correlation of all *MIS*s and nodes in control. The input graph is constructed by replacing the original edges with new edges reflecting control correlations of nodes. We prove that the node adjacent to an input node in input graph will appear in an *MIS*, and the nodes of the same connected component in input graph have the same control type, thus they are either all participate in control or not. Therefore, the emergence of giant connected component in input graph provides a clear topological explanation of the bifurcation phenomenon^12^ in dense networks, and the complex control correlation of nodes of original network can be reduced into a few simple connected components of input graph. Furthermore, we present an efficient method to precisely manipulate the types of any node in control based on its connectivity of input graph. We believe that input graph is important because it (*i*) presents a framework that reveals the inherent correlation of *MIS*s and nodes in control and (*ii*) enables the design and manipulation of a suitable *MIS* of a network under constraints. Ultimately, this will promote the application of network control in real networked systems.

## Results

### Control adjacency and input graph

The dynamics of a linear time-invariant network *G*(*V, E*) is described by:


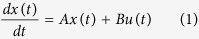


where the state vector ***x***(*t*) = (*x*_1_(*t*), …, *x*_*N*_(*t*))^T^ denotes the value of *N* nodes in the network at time *t, A* is the transpose of the adjacency matrix of the network, *B* is the input matrix that defines how control signals are inputted to the network, and ***u***(*t*) = (*u*_1_(*t*), …, *u*_*H*_(*t*))^T^ represents the *H* input signals at time *t*.

**Figure 1:**
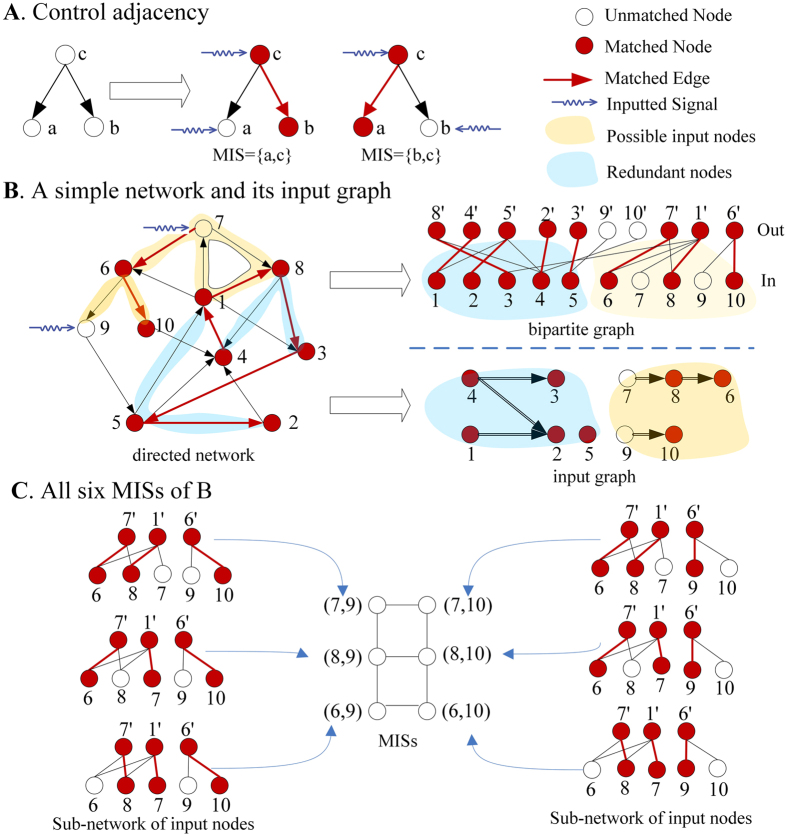
Control adjacency and input graph. (**A**) A simple network with dilation. For a maximum matching {*e*_*cb*_}, input nodes *a* and matched node *b* are control adjacent because they are connected by node *c* with an unmatched edge *e*_*ca*_ and a matched edge *e*_*cb*_, which makes them alternately appear in two *MISs*, {*a, c*} and {*b, c*}; (**B**) Sample directed network (left) and its bipartite graph (right up) and input graph (right down). The bipartite graph are constructed by split nodes of directed network into two separated nodes set in and out, which make a clear representation of control structure. The input graph are built based on control adjacency relationship. The input components (shaded in yellow) contain all possible input nodes and the matched components (shaded in blue) contain all redundant nodes. (**C**) All six *MIS*s of the network shown in (**B**), and the edges are their control adjacent relationship. If an input node of a *MIS* is no longer useable, we can immediate find its substitute node based on input graph. Note that the difference of adjacent *MIS*s are one matched edge and input node, which is the minimal change to original control structure.

To analyze relationship of all nodes in control, we first define the control adjacency of nodes pair: for a network *G* and any maximum matching *M*, node *a* is said to be control adjacent to node *b* if there exists a node *c* connecting *a* and *b* with an unmatched edge *e*_*ca*_ and a matched edge *e*_*cb*_. Control adjacency reveals an important property about control correlation of nodes: a node that is control adjacent to an input node must appear in another *MIS*. For example, in Fig. 1A, input node *a* is control adjacent to node *b*, and the two nodes alternately appear in *MIS* {*a, c*} and *MIS* {*b, c*}. We prove that this property is satisfied by any network, which is called the **Exchange Theorem**, that is: For any *MIS D* of a network *G*, if an input node *n* ∈ *D* is control adjacent to another node *m*, then *D*’ = *D*\{*n*} ∪ {*m*} is also an *MIS* of *G* (see Supplementary Information). This means that a new *MIS D*’ can be obtained from *MIS D* by exchanging a node of *D* with its control adjacent neighbor.

Then, we define the input graph *G*_*D*_(*V, E*_*D*_) based on the control adjacency between nodes, where *V* is the node set, *E*_*D*_ is the edge set and *e*_*ij*_ ∈ *E*_*D*_ if node *i* is control adjacent to node *j* (Fig. 1C). Apparently, the input graph reveals all control relationships of nodes.

The input graph has several potential applications in analyzing controllability of complex networks. We find that the degree of a node in input graph reflects an important property about its substitutability in control. Based on the exchange theorem, for each edge of an input node in input graph, we can find a substitute node and obtain a new *MIS* with only one node replaced. It has important practical value. For example, when an input node of a *MIS* is no longer available due to physical constraint or attack, we can immediate find a new one by replacing the node with one of its neighbor in input graph. The above method makes the minimum change to the original control scheme, which is only one edge. Note that the computational complexity of the above process is only *O*(1), which yields a significantly improved method to obtain a new *MIS* in comparison with the state of the art approach^10^.

### Connected components of input graph

Next we focus on analyzing the connectivity of input graph. Similar to the concept of the path and reachable set in graph theory, we define control path *p* as the node sequence where neighbor nodes are control adjacent. The control-reachable set *C*(*n*) of node *n* is defined as the set of all nodes that are reachable from node *n* through any control path (Fig. 1B). Based on the above definition, we prove the following **Adjacency Corollary**: 1. For any *MIS D* and an input node *n* ∈ *D*, all nodes of *C*(*n*) must be possible input nodes; 2. For any *MIS D*, if node *m* did not belong to any control-reachable set of the input nodes of *D*, then *m* must be a redundant node and never appears in any *MIS* (see Supplementary Information). Therefore, it is easy to conclude that a node is a possible input node if it can be control reachable from an input node of any MIS.

The adjacency corollary show that the control-reachable sets of possible input nodes and redundant nodes will never intersect. Thus, there are only two types of connected components in the input graph: 1. Input Component (*IC*), which contains at least one input node; and 2. Matched Component (*MC*), which contains no input node. We call the two type connected components as control components. Apparently, all nodes of *IC* are possible input nodes and all those of *MC* are redundant nodes.

**Figure 2:**
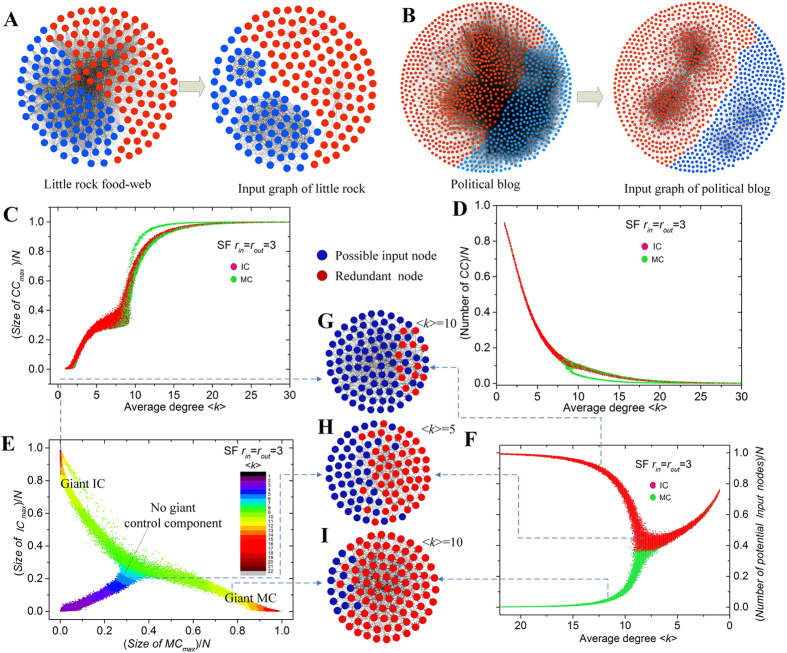
Control components of real and synthetic networks. (**A**,**B**) Real networks and its corresponding control adjacency network. The complex structure of (**A**) *Little rock food-web* and (**B**) *Political blog* are reduced into several simple connected components of input graph. (**C**) The size of the largest control component *CC*_*max*_ versus the average degree <*k*> in scale-free networks with degree exponents *r*_*in*_ = *r*_*out*_ = 3, *N* = 10^4^ and (**D**) the number of control components (*CC*) decreases significantly with increasing <*k*>, which illustrates the emergence of a giant control component; (**E**) two types of giant control components; the input component (*IC*) and matched component (*MC*) cannot coexist in highly connected networks; (**F**) the emergence of a giant control component leads to the bifurcation phenomenon of possible input nodes in dense networks. The majority of nodes of a network with a giant *IC* are possible input nodes, whereas those of a network with a giant *MC* are redundant nodes; (**G**,**H**) three example networks with average degrees of <*k*> = 5 and <*k*> = 10; the type of their giant control component determines the type of the majority of nodes in control.

We analyze the control components of some real networks, and find that the complex control relationships of these networks can be reduced into a few control components of input graphs, i.e., *little rock* (Fig. 2A) and *political blog* networks (Fig. 2B). Furthermore, we find that many real networks have a giant control component in their input graph (Table 1 and Fig. S7), suggesting that the majority of nodes are tightly connected by control adjacency and have the same type in control. The giant control component can either be a giant *IC*, or a giant *MC*.

To further understand the origin of giant control component, we analyze the size of the largest control component of synthetic networks. We found that the size of the largest control component increases with the average degree of a network (Fig. 2C), whereas the number of control components decreases monotonically (Fig. 2D). Therefore, there exists only one giant control component in dense networks (Fig. 2E). The type of a node in control is determined by the type of control component to which it belongs, which depends on whether the control component contains an input node. If the giant control component contain at least one input nodes, most of its nodes will be possible input nodes; and if the giant control component contains no input node, most of its nodes will be redundant nodes. Thus, we can observe the bifurcation phenomenon^12^ (Fig. 2F) that emerges in dense networks. Therefore, the formation of the giant control component in input graph provides a clear explanation for the origin of the bifurcation phenomenon emergent in dense networks.

### Altering the type of nodes in control

Owing to the economical or physical constraints exist in many actual control scenarios, we may need some specified nodes as input nodes. If the node is a possible input node, we can easily find a *MIS* which contain the node. However, if the node is a redundant node, we must alter the structure of the network and turn the node into a possible input node.

**Figure 3:**
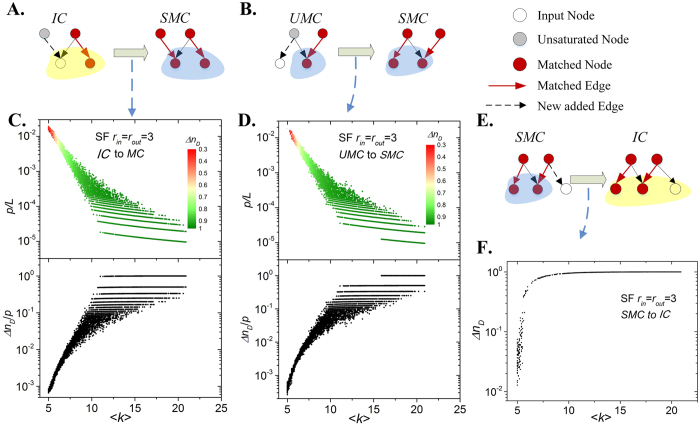
Type alteration of a giant control component in scale-free networks with degree exponents *r*_*in*_ = *r*_*out*_ = 3, *N* = 10^4^. (**A**) Illustration of altering an input component (*IC*) to a saturated matched component (*SMC*). For each input node of an *IC*, we must add an edge pointing from an unsaturated node (node without matched out-edge) to the input node, and the *IC* will turn into an *SMC*; (**B**) Illustration of an alteration of an unsaturated matched component (*UMC*) to an *SMC*. For each unsaturated node of an *MC*, we add an edge from the unsaturated node to an input node, and the *UMC* will turn into an *SMC*; (**C**,**D**). The average degree versus percentage of added edges *p/L* used to alter the control component for *IC* and *UMC* to *SMC*. For large <*k*>, the percentage of added edges significant decreases, and the changed possible input nodes of per edge *Δn*_*D*_/*p* increases rapidly, which indicates that it is easy to alter the giant control component type of dense networks; (**E**) Illustration of an alteration of an *SMC* to an *IC*. We need link an input nodes to *SMC* and it will turn into *IC*; (**F**) Average degree versus density of changed possible input nodes for *SMC* to *IC*. With only one added edge, the control type of most nodes changes in dense network.

Since the nodes of the same control component have same control type, we only need to alter the type of control component in which the target node lies. This problem can be solved by adding new edges to the network. For example, if we link several input nodes to an *MC*, the nodes in the *MC* will be turned into possible input nodes and the *MC* will be turned into an *IC*. Additionally, if we match all input nodes of an *IC*, it will be turned into an *MC* and all nodes in it will be redundant nodes (Fig. 3A,B,E).

Therefore, we present an algorithm to alter the type of the control component (see Method). To quantify the efficiency of the algorithm, we investigate the number of added edges *p* in both *ER-random* and *scale-free* networks. The results (Fig. 3C,D) showed that *p* significantly decreases with the average degree <*k*>, and the proportion of changed possible input nodes *Δn*_*D*_ increases monotonically, which indicates that it is easier to alter the control component of a denser network.

Surprisingly, the giant control component of a few networks can be changed by adding only one edge (Fig. 3E,F). For example, the control type of most nodes of some real networks (e.g. *Facebook* and *Amazon* networks shown in Table 1) can be altered by only one added edge. All these networks have a special giant *MC* in their corresponding input graphs, and the nodes of the *MC* was not linked by any unsaturated node (node without a matched out-edge) in the original network. We call it as a saturated matched component (*SMC*). Therefore, if we link an input node to an *SMC*, most nodes of the *SMC* will be control reachable by the input node and be turned into possible input nodes. However, when an *MC* is linked by one or more unsaturated nodes in original network, which we call it as an unsaturated matched component (*UMC*), we need to match all the unsaturated nodes to change its type. The result show the cost of altering an *IC* to a *UMC* (Fig. 3C) is similar to that of altering a *UMC* to an *IC* (Fig. 3D).

Furthermore, we find that the size of *MIS* significantly decreases after altering the type of the giant control component (Fig. S8), which means that the method can also be used to optimize the controllability of complex networks^19^,^20^.

## Discussion

In summary, we developed the input graph, a fundamental structure that reveals the control relationship of nodes and *MIS*s. Our key finding, that the control adjacent nodes have the same type in control, allows us to reveal the inherent control correlation of nodes, and offers a general mechanism to manipulate the control type of nodes or design a suitable control scheme. Furthermore, networks with a giant control component display a surprising type transition phenomenon in response to well-chosen structural perturbations, which is ubiquitous in dense networks across multiple disciplines.

The input graph presented here is a starting point for deeply investigating the control properties of networks. It paves the way to analyze the properties of all *MIS*s of a network, such as enumerate all *MIS*s^14^, estimate the node capacity in *MIS*s^10^ or find the optimum *MIS* under different control constraints or with specific node property^21^. Furthermore, the structural properties of the input graph, such as the node’s degree and connected component also reveal several important topics on controllability of a network. We believe the other structural properties such as average distance and diameter of input graph, also worth deep investigation for multiple disciplines, such as brain network^22^, protein interaction^18^, and *et al*.

However, besides the input nodes in a *MIS*, there may exist other nodes which also need to be inputted the control signals. These nodes formed a cycle^4^ in the network and cannot accessible from any input nodes in the current *MIS*. Therefore, to fully control the network, a control signal need to be inputted to any node of the cycle, and the signal can be shared with any input node^4^. Furthermore, there may exist more than one input nodes within the same input component. Any of these input nodes can be exchanged with its control reachable node based on exchange theorem. However, if two or more input nodes share same neighbor node in the input graph, they would not be substituted at the same time.

Furthermore, we design an algorithm to alter the control type of most nodes of a network with small structural perturbations, which is the first attempt to convert the control mode^12^,^13^ of a network as far as we know. Many real networks, especially biological network, are incomplete and may have many missing edges. It means that if some new edges are discovered, it may alter the control type of existing nodes dramatically. However, these newly discovered edges will never weaken the performance of our algorithm, because they will only increase the size of the giant connected component of input graph.

Overall, these findings will improve our understanding of the control principles of complex networks and may be useful in controlling various real complex systems, such as drug designs^23^,^24^,^25^, financial markets^16^,^26^ and biological networks^18^,^22^.

## Methods

### Construct input graph

To build an input graph *G*_*D*_(*V, E*_*D*_) of the directed network *G*(*V, E*), we need find all control adjacent edges *E*_*D*_ between nodes. Based on the adjacency corollary, there are no control adjacent relationship between any possible input nodes and redundant nodes. Therefore, the set of edges *E*_*D*_ of input graph are composed by two subsets: the set of edges *E*_*Di*_ between all possible input nodes and the set of edges *E*_*Dr*_ between all redundant nodes.

The edges set *E*_*Di*_ and all possible input nodes *V*_*PD*_ can be found as follows:

1. Find a maximum matching *M* and the corresponding *MIS D*; let the candidate set of all possible input nodes *V*_*PD*_ = *D*;
2. Select a node *n* of *D*, let *D* = *D* − {*n*}; for all in-edges of node *n*, find the corresponding control adjacent neighbors {*c*_1_, *c*_2_, …, *c*_*i*_};
3. Let *D* = *D* + {*c*_1_, *c*_2_, …, *c*_*i*_}*, V*_*PD*_ = *V*_*PD*_ + {*c*_1_, *c*_2_, …, *c*_*i*_} and *E*_*Di*_ = *E*_*Di*_ + {*e*(*n, c*_1_), …, *e*(*n, c*_*i*_)};
4. Repeat step 2 and 3 until *D* is empty.

The edges set *E*_*Dr*_ between all redundant nodes can be found as follows:

1. Let *V*_*temp*_ = *V* − *V*_*PD*_;
2. Select a node *n* of *V*_*temp*_, let *V*_*temp*_ ← *V*_*temp*_ − {*n*};
3. Let the matched in-edge of *n* be *e*(*m, n*), find all out-edge {*e*(*m, c*_1_), …, *e*(*m, c*_*i*_)}of node *m*; let *E*_*Dr*_ = *E*_*Dr*_ + {*e*(*c*_1_, *n*), …, *e*(*c*_*i*_, *n*)} and *V*_*temp*_ ← *V*_*temp*_ *+* {*c*_1_, *c*_2_, …, *c*_*i*_};
4. Repeat step 2 and 3 until *V*_*temp*_ is empty.

Next we analyze the computational complexity of above method. Let *N* is the number of the nodes and *L* is the number of the edges of the directed network *G*(*V, E*). First, the complexity for finding a maximum matching is *O*(*N*^0.5^*L*)^9^. Second, each node requires a breadth first search (*BFS*) process to finding its control reachable set, which computational complexity is *O*(*L*). For the worst case, we need to find the control researchable set for all nodes and the complexity is *O*(*NL*). Therefore, the total computational complexity to building an input graph is O(*NL*).

### Altering an *IC* to a *MC*

For a network *G*(*V, E*), let *B*(*V*_*out*_, *V*_*in*_, *E*) be the corresponding bipartite graph and *IC*_*alter*_ be the target input component. The basic idea of altering an *IC* to a *MC* is to match all input nodes of the *IC* by adding edges. The following are the detailed steps:

1. Find all unmatched nodes *S* corresponding to current maximum matching. Let *S*_1_ = *S* ∩ *V*_*out*_, *S*_2_ = *S* ∩ *V*_*in*_ ∩ *IC*_*alter*_;
2. Select a node *n* ∈ *S*_2_ and a node *m* ∈ *S*_1_; add an edge *e*_*mn*_ to *G*; remove nodes *n* and *m* from *S*_2_ and *S*_1_, respectively;
3. Repeat step 2 until *S*_2_ is empty.

The correctness of method of above method is list as follows. First, we prove that |*S*_1_| ≥ |*S*_2_|. Apparently, Based on the definition of *B*(*V*_*out*_, *V*_*in*_, *E*), |*V*_*in*_| = |*V*_*out*_|. Because every edge of the maximum matching starts with a node of *V*_*out*_ and ends with a node of *V*_*in*_, then |*S*_1_| = |*S* ∩ *V*_*out*_| = |*S* ∩ *V*_*in*_| ≥ |*S* ∩ *V*_*in*_ ∩ *IC*_*alter*_| = |*S*_2_|, which means that for any node of *S*_2_, we can find a corresponding node in *S*_1._ When we add an edge *e*_*mn*_ to *G* in step 3, the matching *M*’ = *M* + *e*_*mn*_ must be a maximum matching of *G*’ = *G* + *e*_*mn*_ because *n* and *m* are not matched by *M*. Therefore, nodes *n* and *m* are matched by *M*’. When we match all input nodes of the *IC*_*alter*_, the *IC*_*alter*_ will be turned into an *SMC*.

### Altering a *MC* to an *IC*

For a network *G*(*V, E*) with a *MC*, let *B*(*V*_*out*_, *V*_*in*_, *E*) be the corresponding bipartite graph. The basic idea of altering a *MC* to an *IC* is to link several input nodes to the *MC* and the *MC* will be turned into an *IC*. For a network with an *UMC*, if we directly link an input node to the *UMC*, the input node will be matched with an unsaturated node of the *UMC*. Therefore, we need first alter the *UMC* to an *SMC* and then turn the *SMC* into an *IC*. The method of altering an *UMC* to an *SMC* is similar to altering an *IC* to an *SMC*, which is as follows:

1. Find all unmatched nodes *S* corresponding to current maximum matching. Let *S*_1_ = *S* ∩ *V*_*in*_, *S*_2_ = *S* ∩ *V*_*out*_ ∩ *UMC*_*alter*_;
2. Select a node *n* ∈ *S*_2_ and a node *m* ∈ *S*_1_; add an edge *e*_*nm*_ to *G*; remove nodes *n* and *m* from *S*_2_ and *S*_1_, respectively;
3. Repeat step 2 until *S*_2_ is empty.

For a network with a giant *SMC*, we only need to link an input node to the *SMC*, and the part which is control reachable by the input node will be turned into an *IC*. Therefore, in order to maximize the size of result *IC*, we need to find the most “influential” node of the *SMC* based on the size of its control-reachable set. The algorithm is listed as follows:

1. Let *SMC*_*alter*_ be the target saturated matched component; compute the size of control-reachable set of nodes in *SMC*_*alter*_, let the node with maximum size be *n*;
2. Let *e*_*mn*_ be the matched edge pointing to node *n*; select an input node *d*, add edge *e*_*md*_ to *G*.

After adding the edge *e*_*md*_, the nodes of control-reachable set of *n* will be turned into possible input nodes because the input node *d* is control adjacent to node *n*. If we want to convert all nodes of an *SMC*, we need to find the minimal input nodes set that can reach all node of the *SMC*. The problem can be solved by a simple greedy algorithm.

## Additional Information

**How to cite this article**: Zhang, X. *et al*. Input graph: the hidden geometry in controlling complex networks. *Sci. Rep.* **6**, 38209; doi: 10.1038/srep38209 (2016).

**Publisher's note:** Springer Nature remains neutral with regard to jurisdictional claims in published maps and institutional affiliations.

## Supplementary Material

Supplementary Information

## Figures and Tables

**Table 1:** Characteristics of the real networks analyzed in the paper.

Type	Name	*N*	*L*	<*k*>	*n*_*MIS*_	*CC*_*max*_	*p*	*Δn*_*D*_
Food Web	StMarks	54	356	13.19	24.07%	38.89%(U)	3.37%	62.96%
	Ythan	135	601	8.90	51.11%	85.19%(I)	10.65%	85.19%
	Mangrove	97	1492	30.76	22.68%	55.67%(I)	1.41%	55.67%
	Florida	128	2106	32.91	23.44%	91.41%(I)	1.38%	91.41%
	Silwood	154	370	4.81	75.32%	84.42%(I)	29.19%	84.42%
	Littlerock	183	2494	27.26	54.10%	34.43%(I)	2.49%	34.43%
Neuronal	C. elegans	306	2345	15.33	18.95%	68.63%(I)	0.90%	68.63%
Transcription	E.Coli	423	578	2.73	72.81%	12.53%(U)	34.78%	1.89%
	TRN-Yeast-1	4441	12873	5.80	96.46%	99.21%(I)	33.04%	99.21%
	TRN-Yeast-2	688	1079	3.14	82.12%	76.60%(I)	41.61%	76.60%
Trust	Prison inmate	67	182	5.43	13.43%	59.70%(U)	2.75%	74.63%
	Slashdot0902	82168	948464	23.09	4.55%	91.23%(I)	0.39%	91.23%
	WikiVote	7115	103689	29.15	66.56%	32.12%(U)	3.60%	26.77%
Electronic circuits	s208a	122	189	3.10	23.77%	17.21%(I)	5.82%	17.21%
	s420a	252	399	3.17	23.41%	9.13%(I)	3.26%	9.13%
	s838a	512	819	3.20	23.24%	5.27%(I)	2.08%	5.27%
Citation	ArXiv-HepTh	27770	352807	25.41	21.58%	48.96%(U)	0.91%	51.89%
	SciMet	3084	10416	6.75	37.48%	52.14%(I)	7.62%	52.14%
	Kohonen	4470	12731	5.70	47.29%	60.25%(I)	14.45%	60.25%
WWW	Political blogs	1224	16718	27.32	34.15%	52.61%(I)	1.46%	52.61%
	NotreDame	325729	1497134	9.19	67.71%	53.90%(I)	9.15%	53.90%
	BerkStan	685230	7600595	22.18	65.69%	57.27%(I)	2.78%	57.27%
	Google	875713	5105039	11.66	36.95%	60.80%(I)	3.97%	60.80%
	Stanford	281903	2312497	16.41	35.91%	63.50%(I)	2.83%	63.50%
Internet	p2p-1	10876	39994	7.35	55.20%	90.58%(I)	14.88%	90.58%
	p2p-2	8846	31839	7.20	57.78%	90.55%(I)	15.52%	90.55%
	p2p-3	8717	31525	7.23	57.74%	91.75%(I)	15.58%	91.75%
Organizational	Consulting	46	879	38.22	4.35%	97.83%(I)	0.23%	97.83%
Social communication	UClonline	1899	20296	21.38	32.33%	79.94%(I)	2.84%	79.94%
Product co-purchasing	Amazon0302	262111	1234877	9.42	3.23%	49.55%(U)	0.30%	74.58%
	Amazon0312	400727	3200440	15.97	3.52%	83.61%(S)	0.00003%	75.74%
	Amazon0505	410236	3356824	16.37	3.62%	91.35%(I)	0.44%	91.35%
	Amazon0601	403394	3387388	16.79	2.04%	75.90%(U)	0.21%	90.73%
Social network	twitter_combined	81306	1768149	43.49	19.39%	79.40%(I)	0.88%	79.40%
	Facebook_0	347	5038	29.04	5.48%	86.46%(S)	0.02%	81.84%
	Facebook_107	1912	53498	55.96	45.92%	54.08%(S)	0.001%	53.24%
	Facebook_348	572	6384	22.32	61.01%	38.64%(S)	0.02%	38.29%

For each network, we show its type, name, number of nodes (*N*) and edges (*L*), density of input nodes *n*_*MIS*_, size and type of the largest control component *CC*_*max*_, in which *I, U* and *S* denote *IC, UMC* and *SMC* respectively, the proportion of edges (*p*) that is added into the network to change the type of *CC*_*max*_ and the density of changed possible input nodes (*Δn*_*D*_) after adding edges.
