# Supplementary material for: Input graph: the hidden geometry in controlling complex networks
Source: Sci Rep. 2016 Nov 30;6:38209. doi: 10.1038/srep38209 (PMC5128914; doi:10.1038/srep38209)
Supplement: Supplementary Information [file srep38209-s1.pdf]

Input graph: the hidden geometry in controlling complex networks

Xizhe Zhang [1], Tianyang Lv [2,3], Yuanyuan Pu[1]

[1] （School of Computer Science and Engineering, Northeastern University, Shenyang110819, China）

[2] （College of Computer Science and Technology, Harbin Engineering University, Harbin 150001, China)

[3] （IT Center, National Audit Office, Beijing 100830, China)

Supplementary Information:

Materials and Methods

Figures S1-S8

Tables S1-S2

.

Materials and Methods

1. Background

We consider a linear time-invariant system $G(A, B)$, whose states are determined by the following equations [1]:

$$\frac{dx(t)}{dt} = Ax(t) + Bu(t) \tag{1}$$

where the state $\mathbf{x}(t)=(x_1(t),\ldots, x_N(t))^T$ denotes the value of all nodes at time t; A is the transpose of the adjacency matrix; $\boldsymbol{u}(t)=(u_1(t),\ldots,u_M(t))^T$ is the input signal; B is the input matrix that defines how control signals are inputted into the system.

The above system $G(A, B)$ is considered to be controllable [2-3] if it can be driven from the initial state to any admissible final state, which can be determined by Kalman's controllability rank condition [3], that is, the network is controllable if and only if the following matrix has full rank:

$$C = (B,\ AB,\ A^2B,\ldots,\ A^{N-1}B) \tag{2}$$

In many real control scenarios, the edge weight of a network is often unknown or time-variant. To overcome this difficulty, Lin [4] introduced structural controllability, which considers a network where only the structure of the zero and nonzero elements is known. If a network is structural controllable, it remain structural controllable for almost all weight values. To fully control the network, we need to input external signals to the nodes of a network. Because one input signal can be connected to multiple nodes, we call those nodes which do not share input signals as input nodes. The input nodes are also called driver nodes [5-8].The Minimum set of independent input nodes that is used to fully control a network is called **M**inimum **I**nput nodes **S**et (*MIS*) [5-8].

An *MIS* can be determined by the maximum matching of a network [9], the unmatched nodes corresponding to any maximum matching are input nodes. Liu et.al [5] found that the input nodes tends to avoid high degree nodes and the size of an *MIS* is mainly determined by the degree distribution of a network. Ruths [6] quantified the node composition of an *MIS* and found that most real networks form three well-defined clusters. Menichetti [7] found that the fraction of input nodes is primarily determined by low in-degree and low out-degree nodes.

However, the maximum matchings of a network are generally not unique, as are *MIS*s. Jia [8] classified the nodes based on their participation in all *MIS*s: 1. critical input node, which appear in all *MIS*s; 2. redundant node, which never appear in any *MIS*; 3. intermittent input node, which appears in one or more *MIS*s. They found that the density of intermittent input nodes exhibit a surprising bifurcation phenomenon in dense networks, in which the majority of nodes are either intermittent input nodes or redundant nodes. However, because enumerating all possible maximum matching is a #P problem [10], it is very difficult to analyze all *MIS*s of large-scale networks. Jia et.al [11] analyzed the bifurcation phenomenon based on the greedy leaf removal (GLR) procedure [12]. They found the types of the core determined the type of majority nodes in control [8]. However,

we still lack knowledge regarding the correlation of all input nodes and the method to alter the type of nodes in control.

2. Maximum matching

In this section we will introduce some basic concepts and theorems about maximum matching. The following contents can be found in the textbook of graph theory [13].

A graph B (V_1, V_2, E) is called a bipartite graph if there are no edge connect nodes of V_1 and V_2. A set of edges in B (V_1, V_2, E) is called a matching M if no two edges in M have a node in common. A node v_i is said to be matched by M if there is an edge of M linked to v_i; otherwise, v_i is unmatched. A path P is said to be M-alternating if the edges of P are alternately in and not in M. An M-alternating path P that begin and ends at the unmatched nodes is called an M-augmenting path. The maximum matching is a matching with the maximum number of edges of the graph.

For any matching M, if there exist an M-augmenting path P, we can obtain a larger matching M' by the symmetric difference of M and $E(P)$, that is, $M' = M\Delta E(P) = (M\backslash E(P)) \cup (E(P)\backslash M)$. Therefore, the maximum matching can be found by searching the M-augmenting paths of the network, which are proved by the following theorem:

Berge Theorem [14]: Let M be a matching of bipartite graph B, M is a maximum matching if and only if there exist no M-augmenting path in B corresponding to M.

A direct inference of above theorem is: for a given maximum matching M, if P is an M-alternating path which start or end by one unmatched node, the symmetric difference of M and $E(P)$ will result a different maximum matching M'.

Next we will introduce the maximum matching of a directed network which used to analyze the structural controllability [9]. Consider a directed network $G(V,E)$, where $V(G)$ is the node set and $E(G)$ is the edge set. To analyze the structural controllability, we first define its corresponding bipartite graph $B(V^{out},V^{in},E)$ as the follows: V^{out} is the set of nodes with out-edges in V, and V^{in} is the set of nodes with in-edges in V. A directed link e_{ij} corresponds to a connection between node i of V^{out} and node j of V^{in} in the bipartite graph. (see Figure S1 for a detailed example).

An *MIS* can be obtained by computing the maximum matching of $B(V^{out},V^{in},E)$, the unmatched nodes in V^{in} are input nodes [9], which can be inputted external control signals. The unmatched nodes in V^{out} are called unsaturated nodes. Because $B(V^{out},V^{in},E)$ is constructed based on the directed network $G(V,E)$, therefore, for directed network G, the input nodes have no matched in-edge and the unsaturated nodes have no matched out-edge.

3. Theorem

3.1 Exchange Theorem

Definition 1: Control adjacency: For a network *G* and its maximum matching *M*, consider nodes *a* and *b*, if there exist a node *c*, an unmatched edge (*c*,*a*) and a matched edge (*c*,*b*), we say that *a* is control adjacent to *b*.

Exchange Theorem: For a network *G* and one of its *MIS D*, if there exist a input node $a \in D$ with non-zero in-degree $k_{in}(a) \neq 0$, then another node *b* which is control adjacent to node *a* must exist, and $D' = D \backslash \{a\} \cup \{b\}$ is also an *MIS* of *G*.

Proof: Let *M* be the maximum matching corresponding to *D*. Because $k_{in}(a) \neq 0$, let $e_1=(c,a)$ be an in-edge of node *a*. First, we prove that node *c* has a matched out-edge. If *c* has no matched out-edges, *c* must be an unmatched node, which means that edge $e_1=(c,a)$ connects two unmatched nodes *c* and *a* (*a* is a input node of *D*). Therefore, based on the definition of matching, $M \cup \{e_1\}$ is also a matching of G. This contradicts to the fact that *M* is a maximum matching of *G*. Therefore, *c* must have a matched out-edge, denoted as $e_2=(c,b)$. By definition 1, the node *a* is control adjacent to node *b*.

The next step is show that $D' = D \backslash \{a\} \cup \{b\}$ is an *MIS* of *G*, that is to prove that $M' = M \backslash \{e_2\} \cup \{e_1\}$ is another maximum matching of *G*. For maximum matching *M*, we already know that node *a* have no matched in-edge (because it is a input node), $e_1=(c,a)$ is a unmatched edge and $e_2=(c,b)$ is a matched edge. Therefore, let $e_1=(c,a)$ be the matched edges and $e_2=(c,b)$ be the unmatched edge, according to the definition of matching, $M' = M \backslash \{e_2\} \cup \{e_1\}$ is still a matching of *G* (see Figure.S2A). Clearly, *M*' is a maximum matching because it have same number of edges as maximum matching *M*. Therefore, the unmatched nodes $D' = D \backslash \{a\} \cup \{b\}$ that corresponds to *M*' is an *MIS* of network *G*. The proof is complete.

Inference 1: A node appears in all *MIS*s if and only if it has no in-edge.

Proof: Sufficiency. Based on exchange theorem, if node *n* appeared in all *MIS*s, there must not exist a node that is control adjacent to *n*, which means that the $k_{in}(n)=0$.

Necessity. Suppose that $k_{in}(n)=0$. Because no edge point to node *a*, it is unmatched for any maximum matchings. Therefore, node *n* appears in all *MIS*s.

Inference 2: The number of control adjacent neighbors of input nodes *n* equals its in-degree.

Proof: Based on exchange theorem, for each in-edge of node *n*, there must exist a control adjacent node. Because the matched edges do not share nodes, therefore, each in-edge of *n* correspond to different control adjacent node. The proof is complete.

3.2 Input graph and control components

Definition 2: Input graph: For a network *G*(*V*,*E*) and one of its maximum matching *M*, the input graph $G_D(V,E_D)$ are defined based on the control adjacent relationship of nodes set *V*, where E_D is the edge set and $e_{ij} \in E_D$ if node *i* is control adjacent to node *j*.

Definition 3: Control path: For an edge sequence $P(e_1,e_2,\ldots,e_k)$ of *G*, we say that *P* is a control

path if and only if for any two adjacent edges $e_i(x,a)$ and $e_{i+1}(x,b)$ in $P(e_1,e_2,\ldots,e_k)$, node a is control adjacent to b. We say that node a is control reachable to node b if there exist a control path connecting them.

Definition 4: Control-reachable set: All nodes that are control reachable from input node a are called the control-reachable set of a, denoted as $C(a)$. Note that $a \in C(a)$. The control-reachable set of *MIS D* is the union of its input nodes' control-reachable set, that is, $C(D) = \bigcup_{n \in D} C(n)$. For a matched node b, the control-reachable set $C(b)$ is defined as the set of nodes that is control reachable to b.

For nodes n and m, $C(n)$ and $C(m)$ are connected if $C(n) \cap C(m) \neq \emptyset$. The control-reachable set of nodes of a network may connect with each other, and form the following connected components:

Definition 5: Control component: For any maximum matching of network G, the maximal connected sets of the control-reachable set of G are called the control components of G.

The control component can be classified as the following: 1. an input component (*IC*), which contains at least one input node (node without any matched in-edge); 2. a matched component (*MC*), which does not contain any input node.

Lemma 1: A control component cannot both contain an input node and link by an unsaturated node of G.

Proof: For network G and maximum matching M, let C be a control component and contains an input node n. Let node m be an unsaturated node. According to the definition of unsaturated node and input node, nodes n have no matched in-edge and node m have no matched out-edge.

Suppose node m is connected to a node of C, which have two following cases (Figure.S2B):

1. Node m is connected to input node $n \in C$. Because n and m are both unmatched nodes in the corresponding bipartite graph of G, that means that $e(m,n) \notin M$. Therefore, $M' = M + e(m,n)$ is another matching of G. That contradict to the fact that M is a maximum matching because $|M'|>|M|$.

2. Node m is connected to another matched node $k \in C$. Based on the definition of control component, there must exist an input node j which control reachable to k. Let the control path from j to k be $p(n,k)$, clearly, $p(j,k)+e(m,k)$ is an M-augmenting path because the node m and node j are both unmatched in the corresponding bipartite graph of G. That means that M is not a maximum matching based on Berge theorem [12]. This leads to a contradiction. Therefore, a control component cannot both contain an input node and link by an unsaturated node.

3.3 Adjacency Corollary

Because the input graph is constructed based on the maximum matching of the network, different maximum matchings may result different input graphs. However, the types of nodes in control and the types of control components are remain same for any maximum matching, which is proved by the following theorem.

Adjacency Corollary 1: For any *MIS D* and input node $a \in D$, all nodes of $C(a)$ are possible input nodes.

Proof: Let the maximum matching related to *MIS D* be *M*. For any node b∈*C*(*a*), based on the definition of a control-reachable set, there must exist a path P_{ab} which starts with unmatched node *a* and ends with matched node *b*. Based on the definition of section 2, P_{ab} is an *M*-alternating path.

Let *M*' be the symmetric difference of *M* and $E(P_{ab})$, that is, $M^{'} = M\Delta E(P_{ab})$. Based on the inference of Berge theorem mentioned in section 2, *M*' is another maximum matching because P_{ab} is an *M*-alternating path. Clearly, node *b* is not matched by *M*' because it is matched by *M* (Figure.S2C). Therefore, node *b* is an input node corresponding to maximum matching M'. The proof is complete.

Before we prove the Adjacency Corollary 2, we give the following property [15] about the symmetric difference of two maximum matching:

Property 1 [15]: For two different maximum matching *M* and *M*', each connected component of the symmetric difference $M\Delta M' = (M\backslash M') \cup (M'\backslash M)$ is one of the following (see Figure.S3):

(1) An isolated node.

(2) An even cycle with edges alternatively in *M\M'* and *M'\M*, or

(3) A path whose edges are alternatively in *M\M'* and *M'\M*.

Adjacency Corollary 2: For any *MIS D*, if node $b \notin C(D)$, *b* must be a redundant node.

Proof: Let *M* be the maximum matching corresponding to *MIS D*. Based on the definition of control reachable set, node *b* is matched by *M* and has one matched in-edges $e_{nb} \in M$ because $b \notin C(D)$. Suppose that *b* appears in another *MIS D*' and *M*' is the corresponding maximum matching. Because we try to prove node *b* is not a possible input node, therefore, we only consider the in-edge of node *b* in the following proof. Clearly, all in-edges of node *b* are not matched corresponding to *M*'.

Now we consider the symmetric difference $M\Delta M'$. Let *CP*(*b*) be the connected component of the symmetric difference $M\Delta M'$ which contained node *b*. Based on Property 1, the symmetric difference of two maximum matchings have three cases. Note that node *b* is matched by *M* and is not matched by *M*'. Therefore, *CP*(*b*) is not an isolated node, or an even cycle (node *4* and *5* in Figure.S4D), or a path (node *6* in Figure.S4D), because node *b* are matched by both *M* and *M*' in all three cases. Therefore, *CP*(*b*) can be only *C*1 (node 2 or 3) in Figure.S4D: path *P* with an even length that starts with node *b* and ends with another node *n* of V^{in}.

Next we show that the end node *n* of path *CP*(*b*) is an unmatched node corresponding to maximum matching *M*. Because path *CP*(*b*)$\in M\Delta M'$, the edges of path *CP*(*b*) alternately appears in *M* and *M*'. Because the length of *CP*(*b*) is even, and node *b* is matched by *M*, it is easy to conclude that node *n* is not matched by *M*.

Finally, we prove that node *n* is control reachable to node *b*. Because node *b* is matched by *M*, *n* is not matched by *M* and the edges of *CP*(*b*) alternately appears in and out *M*. Therefore, based on the definition of control reachable set, node *n* is control reachable to node *b* corresponding to maximum matching *M*, that is $b \in C(a) \in C(D)$. This leads to a contradiction. Therefore, *b* never appears in any *MIS* and must be a redundant node.

Based on the adjacency corollaries 1 and 2, we obtain the following important inference：

Inference 4: The control-reachable set of any *MIS D* is the union of all *MIS*s of a network.

Proof: Based on the theorem 3 and 4, the proof is trivial.

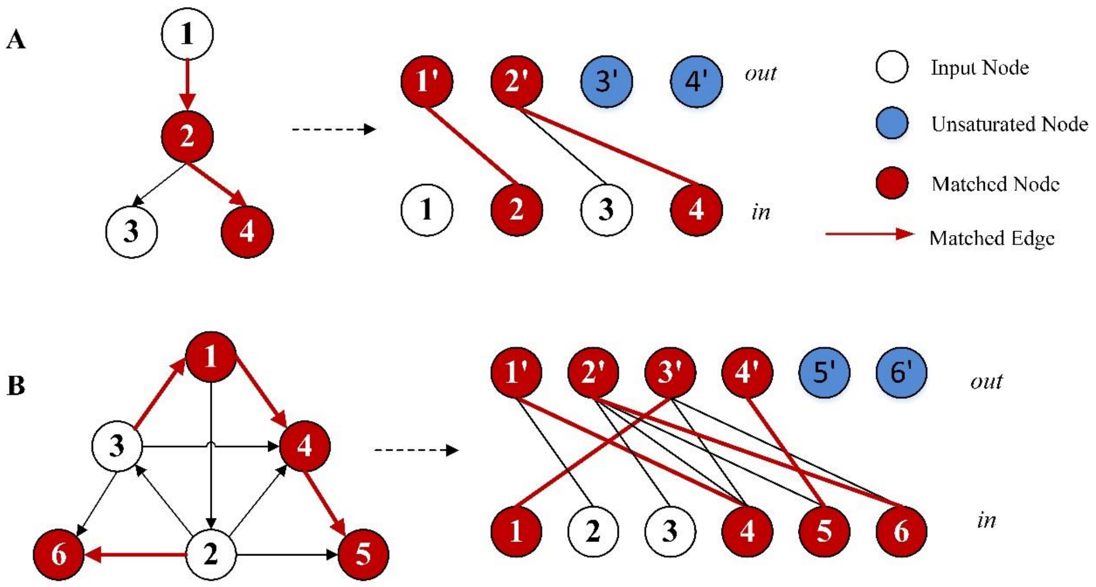

Figure S1: Two sample directed networks and their corresponding bipartite graphs. The red nodes and edges are matched nodes and matched edges of the maximum matchings. A node *n* of a sample network corresponds to two nodes of the bipartite graphs that belong to the *in* set and the *out* set of nodes respectively, recorded as *n* and *n*'. An edge $a_{n,m}$ of a sample network corresponds to an edge $e_{n',m}$ of the bipartite graph, where *n'* belongs to the *out* set and *m* belongs to the *in* set.

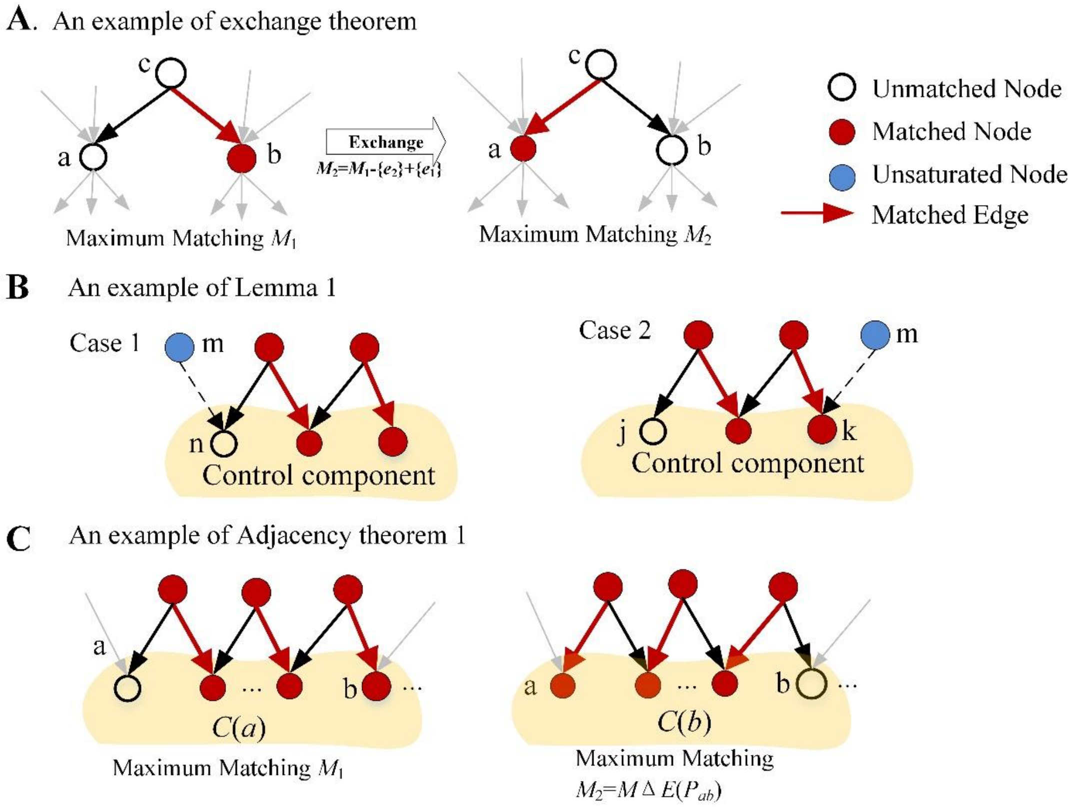

Figure S2: Some examples used in the proof of theorems. **A**. An example network and one of its maximum matching, node *a* is an input node and *b* is a matched node. If we exchange the matched edge e_{cb} and the unmatched edge e_{ca}, we will get a new *MIS* in which node *b* is an input node and node *a* is a matched node; **B**. An example network used in the proof of Lemma 1. Case1: If unsaturated node *m* is connected to node *n*, edge e_{mn} will be a matched edge because *m* and *n* are both unmatched by current maximum matching; Case 2: if node *m* connected to a matched node *j*, then path P_{mj} will be a *M*-augmenting path because the node *m* and node *j* are both unmatched; **C**. An example network used in the proof of Adjacency Corollary 1. For a maximum matching M_1, if node *b* is control reachable by input node *a*, then P_{ab} must be an *M*-alternating path. The symmetric difference of *M* and $E(P_{ab})$ M_2 is also a maximum matching, and the node b is not matched by M_2.

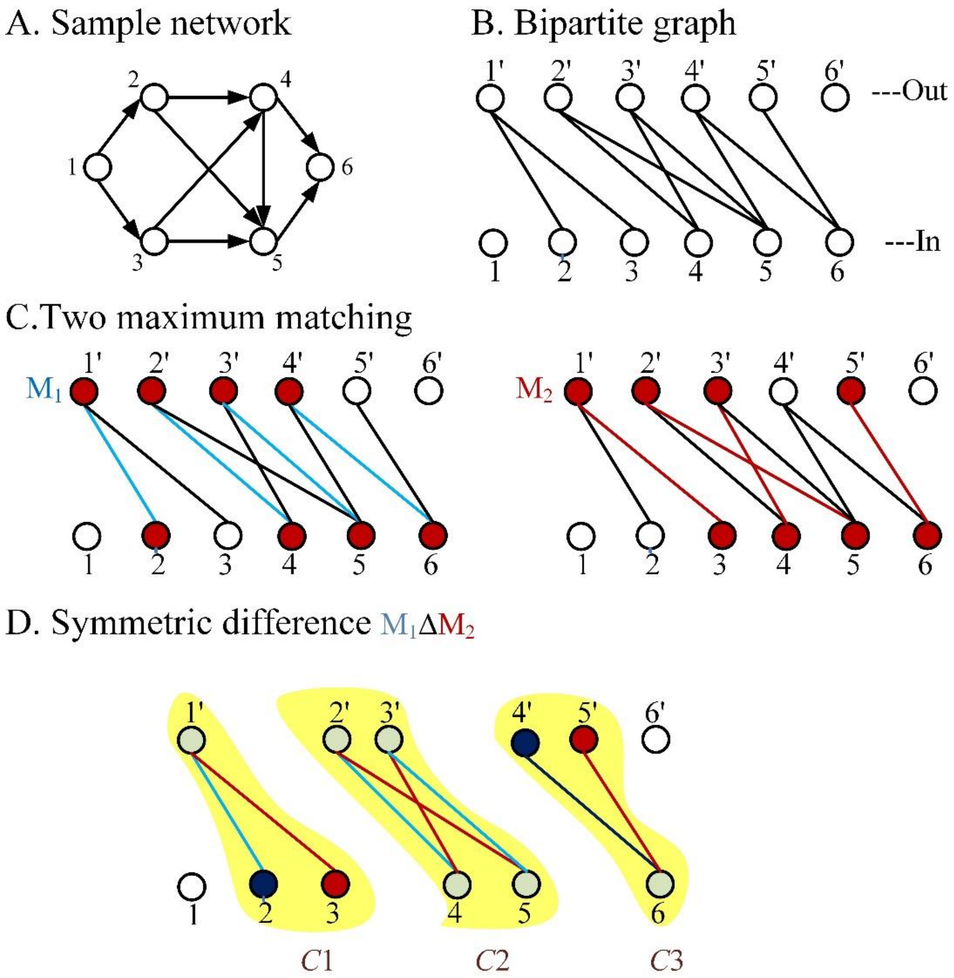

Figure S3: Symmetric difference of two maximum matchings of a sample network. (a) Sample network and its bipartite graph in (b); (c) two maximum matchings M_1 and M_2 of (b); (d) Symmetric difference of M_1 and M_2. There are three cases of its connected components: *C*1, *C*2 and *C*3. *C*1 and *C*3 are the paths whose edges are alternatively appear in $M_1 \backslash M_2$ and $M_2 \backslash M_1$. *C*2 is the even cycle with edges alternatively in $M_1 \backslash M_2$ and $M_2 \backslash M_1$. If M_1 and M_2 are maximum matchings corresponding to two different *MDS*s, the connected components of symmetric difference of M_1 and M_2 will only contain *C*1 type component.

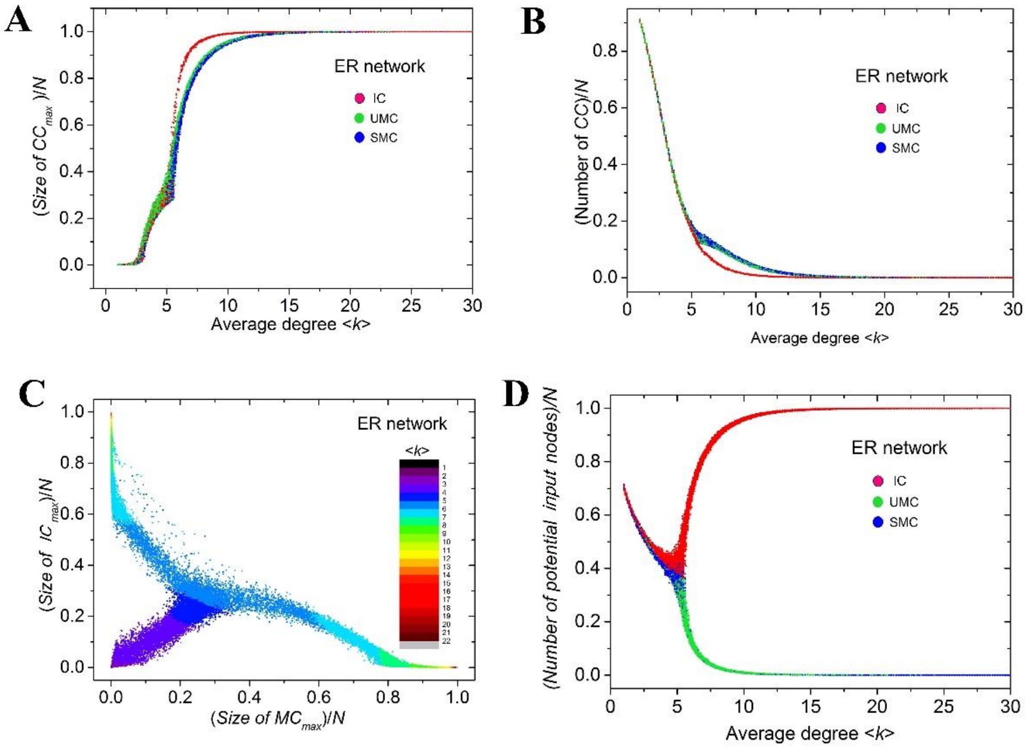

Figure S4: Control component of *ER* networks. The number of nodes N=10^4. (A). The size of the largest control component CC_{max} versus the average degree <*k*> in *ER* networks and (B) the number of control components (*CC*) decreases significantly with the increasing <*k*>, which illustrates the emergence of a giant control component; (C) two type of the giant control components, the input component (*IC*) and the matched component (*MC*) cannot coexist in the highly connected networks; (D) the emergence of the giant control component leads to the bifurcation phenomenon of possible input nodes in dense networks. The majority of nodes of a network with a giant *IC* are possible input nodes, whereas those of a network with a giant *MC* are redundant nodes.

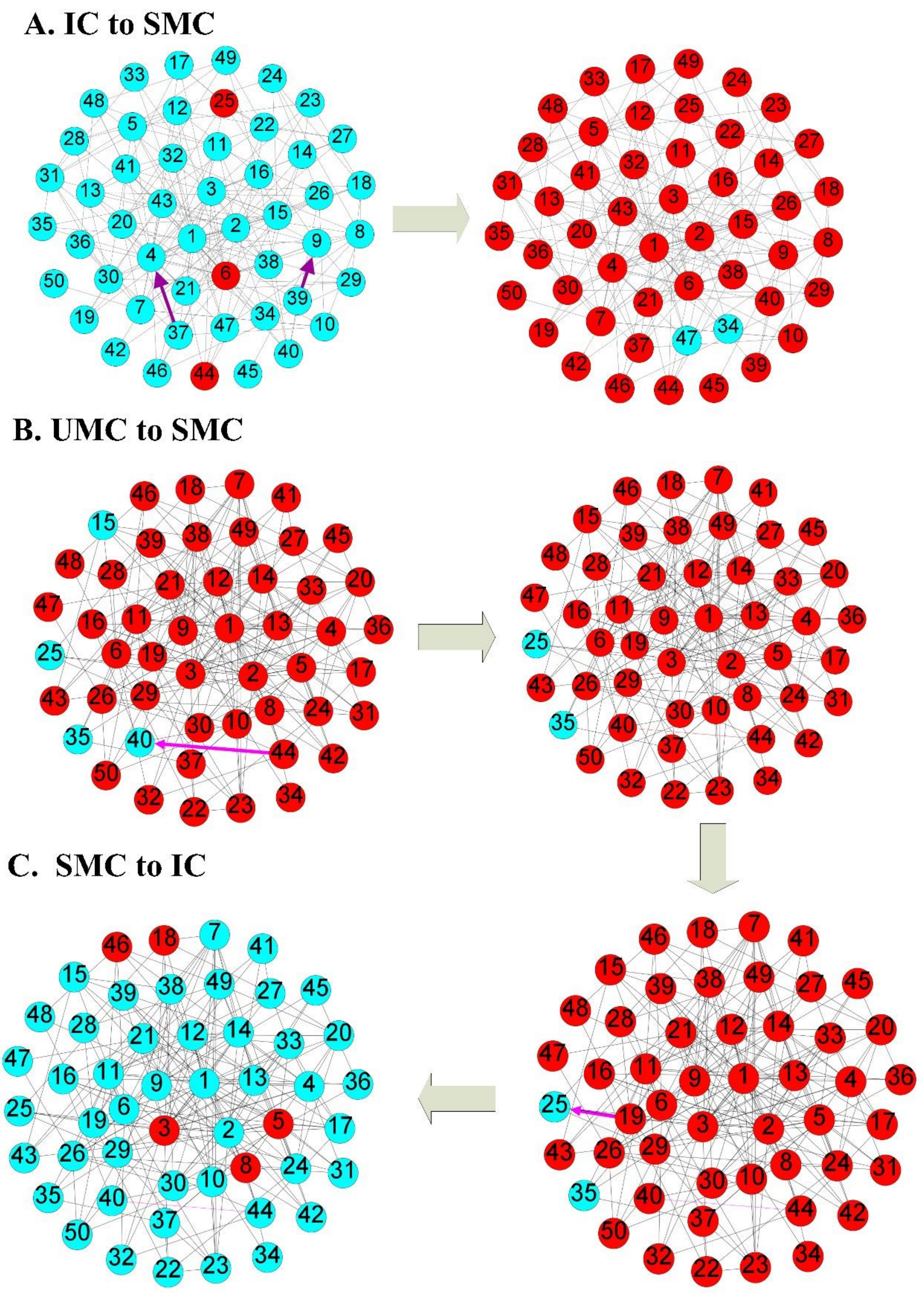

Figure. S5 | Illustration of altering the type of the giant control component of sample networks. The number of nodes N=50. (**A**) Alternation of an *IC* to an *SMC* by adding two edges. After add the edges e(37,4) and e(39,9), most possible input nodes (blue nodes) are turned into redundant nodes (red nodes); (**B**) Alternation of an *UMC* to an *SMC* by adding one edge. After adding the edge e(44,40), the *UMC* is turned into an *SMC*, and (**C**) After adding the edge e(19,25), most redundant nodes (red nodes) are turned into possible input nodes (blue nodes), and the giant control component is turned into a giant *IC*.

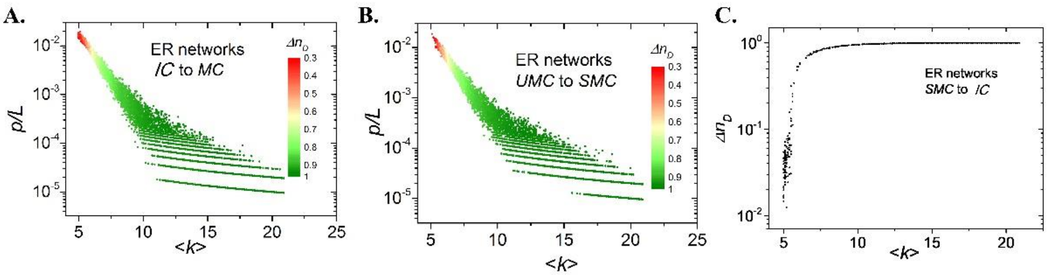

Figure. S6 | Percentage of added edges *p/L* used to alter the giant control component versus average degree <*k*> of ER networks. The number of nodes N=10^4. (**A**) When one alter an *IC* to an *UMC*, the percentage of added edges significant decreases with increasing <*k*>, and the number of changed possible input nodes of each added edge $\Delta n_D/p$ increases rapidly; (**B**) When one alters an *UMC* to an *SMC*, the percentage of added edges used to alter the giant control component is similar to the case that one alters an *IC* to an *UMC*; (**F**) When one alters an *SMC* to an *IC*, the control type of most nodes in a dense network will be changed by adding only one edge.

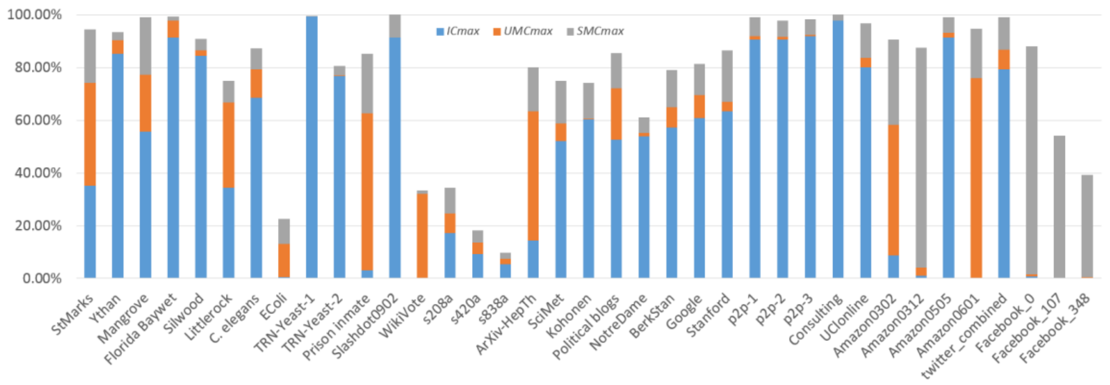

Figure.S7 | Control component of real networks. The fractions of IC_{max} (blue), UMC_{max} (orange) and SMC_{max} (grey) for the real networks named in Table 1. Note that we only counted the maximum control component of each type, thus the sum of fractions may be less than 100%.

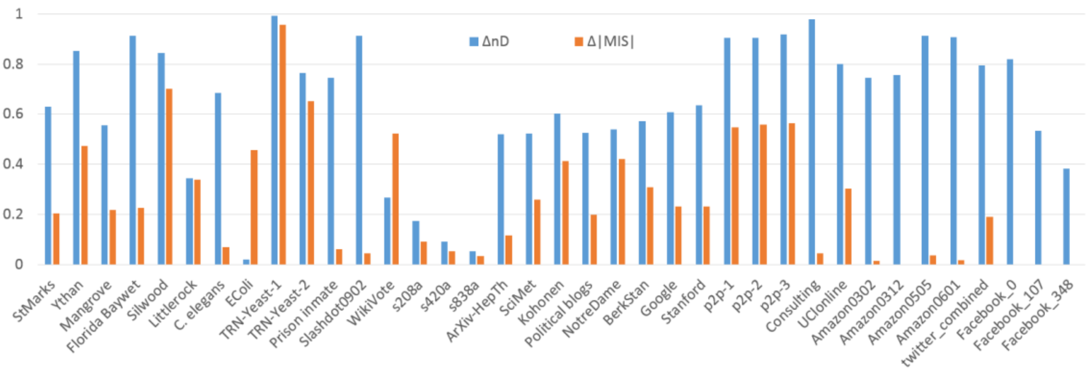

Figure.S8 | The changed fraction of possible input nodes (blue) and the size of *MIS* (orange) after type transition of real networks shown in Table 1. The size of *MIS* significant decreases after type transition, which makes the networks easier to be controlled.

Table S1. Real networks analyzed in this paper. For each network, we show its type, name, number of nodes (N) and edges (L), and brief description.

Type	Name	*N*	*L*	Description
**Food Web**	StMarks[16]	54	356	Food Web in St Marks national wildlife refuge.
	Ythan[17]	135	601	Food Web in Ythan Estuary.
	Mangrove[18]	97	1492	Food Web in Mangrove Estuary, Wet Season
	Florida[18]	128	2106	Food Web in Florida Bay
	Silwood[19]	154	370	Food Web in Silwood Park
	Littlerock[20]	183	2494	Food Web in Little Rock lake.
**Neuronal**	C. elegans[21]	306	2345	Neural network of C. elegans
**Transcription**	E.Coli[22]	423	578	Transcriptional regulation network of Escherichia coli
	TRN-Yeast-1[23]	4441	12873	Transcriptional regulatory network of *S. cerevisiae*
	TRN-Yeast-2[24]	688	1079	Transcriptional regulatory network of *S. cerevisiae* (compiled by different group).
**Trust**	Prison inmate[25,26]	67	182	Social networks of positive sentiment
	Slashdot [27]	82168	948464	Social network (friend/foe) of Slashdot users
	WikiVote[28]	7115	103689	Who-vote-whom network of Wikipedia users
**Electronic circuits**	s208a[29]	122	189	Electronic sequential logic circuit.
	s420a[29]	252	399	
	s838a[29]	512	819	
**Citation**	ArXiv-HepTh[30]	27770	352807	Citation network of high energy physics theory in arXiv (1993-2003)
	SciMet[31]	3084	10416	Citation network in Scientometrics (1978-2000)
	Kohonen[32]	4470	12731	Citation network with topic self-organizing maps
**WWW**	Political blogs[33]	1224	16718	Hyperlinks between weblogs on US politics
	NotreDame[34]	325729	1497134	Web pages from University of Notre Dame
	BerkStan[27]	685230	7600595	Web pages from University of Notre Dame berkely.edu and stanford.edu (2002)
	Google[27]	875713	5105039	Web pages from Google Programming Contest
	Stanford[27]	281903	2312497	Web pages from Stanford University
**Internet**	p2p-1[35]	10876	39994	Gnutella peer-to-peer file sharing network (2002.08.04)
	p2p-2[35]	8846	31839	Gnutella peer-to-peer file sharing network (2002.08.05)
	p2p-3[35]	8717	31525	Gnutella peer-to-peer file sharing network (2002.08.06)
**Organizational**	Consulting[36]	46	879	Social network from a consulting company.
**Social communication**	UClonline[36]	1899	20296	Online message network of students at UC, Irvine.
**Product co-purchasing**	Amazon0302[37]	262111	1234877	Amazon product co-purchasing network (2003.0302)
	Amazon0312[37]	400727	3200440	Amazon product co-purchasing network (2003.0312)
	Amazon0505[37]	410236	3356824	Amazon product co-purchasing network (2003.0505)
	Amazon0601[37]	403394	3387388	Amazon product co-purchasing network (2003.0601)
**Social network**	twitter_combined[38]	81306	1768149	Social circles from Twitter (combined 973 egonets)
	Facebook_0[38]	347	5038	Social circles of user 0 from Facebook

	Facebook_107[38]	1912	53498	Social circles of user 107 from Facebook
	Facebook_348[38]	572	6384	Social circles of user 572 from Facebook

Table.S2. Characteristics of the real networks analyzed in this paper. For each network, IC_{max}, UMC_{max}, SMC_{max} are the relative size of the largest input, the largest unsaturated matched and the largest saturated matched control component, respectively; p_e is the percentage of edges used to alter the type of the largest control component. $\boldsymbol{n_{D1}}$ and MIS_1 are the number of possible input nodes and the size of *MIS* before the type transition; $\boldsymbol{n_{D2}}$ and MIS_2 are the number of possible input nodes and the size of *MIS* after the type transition.

Type	Name	IC_{max}	UMC_{max}	SMC_{max}	p_e	n_{D1}	n_{D2}	MIS_1	MIS_2
**Food Web**	StMarks	35.19%	38.89%	20.37%	3.37%	37.04%	100.00%	24.07%	3.70%
	Ythan	85.19%	5.19%	2.96%	10.65%	89.63%	4.44%	51.11%	3.70%
	Mangrove	55.67%	21.65%	21.65%	1.41%	56.70%	1.03%	22.68%	1.03%
	Florida Baywet	91.41%	6.25%	1.56%	1.38%	92.19%	0.78%	23.44%	0.78%
	Silwood	84.42%	1.95%	4.55%	29.19%	93.51%	9.09%	75.32%	5.19%
	Littlerock	34.43%	32.24%	8.20%	2.49%	56.28%	21.86%	54.10%	20.22%
**Neuronal**	C. elegans	68.63%	10.78%	7.84%	0.90%	81.05%	12.42%	18.95%	12.09%
**Transcription**	EColi	0.47%	12.53%	9.46%	34.78%	73.05%	74.94%	72.81%	27.19%
	TRN-Yeast-1	99.21%	0.001%	0.09%	33.04%	99.91%	0.70%	96.46%	0.70%
	TRN-Yeast-2	76.60%	0.44%	3.63%	41.61%	94.91%	18.31%	82.12%	16.86%
**Trust**	Prison inmate	2.99%	59.70%	22.39%	2.75%	16.42%	91.04%	13.43%	7.46%
	Slashdot0902	91.23%	0.002%	8.75%	0.39%	91.23%	0	4.55%	0
	WikiVote	0.03%	32.12%	1.25%	3.60%	66.59%	93.37%	66.56%	14.17%
**Electronic circuits**	s208a	17.21%	7.38%	9.84%	5.82%	33.61%	16.39%	23.77%	14.75%
	s420a	9.13%	4.37%	4.76%	3.26%	32.94%	23.81%	23.41%	18.25%
	s838a	5.27%	2.15%	2.34%	2.08%	32.62%	27.34%	23.24%	19.92%
**Citation**	ArXiv-HepTh	14.44%	48.96%	16.71%	0.91%	33.98%	85.87%	21.58%	10.06%
	SciMet	52.14%	6.55%	16.28%	7.62%	64.40%	12.26%	37.48%	11.74%
	Kohonen	60.25%	0.27%	13.62%	14.45%	66.91%	6.67%	47.29%	6.13%
**WWW**	Political blogs	52.61%	19.53%	13.40%	1.46%	66.91%	14.30%	34.15%	14.22%
	NotreDame	53.90%	1.16%	6.01%	9.15%	87.04%	33.14%	67.71%	25.64%
	BerkStan	57.27%	7.77%	13.99%	2.78%	73.21%	15.94%	65.69%	34.83%
	Google	60.80%	8.84%	11.68%	3.97%	73.58%	12.78%	36.95%	13.83%
	Stanford	63.50%	3.56%	19.36%	2.83%	72.13%	8.63%	35.91%	12.66%
**Internet**	p2p-1	90.58%	1.34%	7.13%	14.88%	91.11%	0.52%	55.20%	0.47%

	p2p-2	90.55%	0.97%	6.15%	15.52%	92.64%	2.09%	57.78%	1.92%
	p2p-3	91.75%	0.76%	5.79%	15.58%	93.25%	1.50%	57.74%	1.40%
**Organizational**	Consulting	97.83%	0	2.17%	0.23%	97.83%	0	4.35%	0
**Social communication**	UClonline	79.94%	3.84%	12.95%	2.84%	81.89%	1.95%	32.33%	1.95%
**Product co-purchasing networks**	Amazon0302	8.76%	49.55%	32.32%	0.30%	17.74%	92.32%	3.23%	1.79%
	Amazon0312	0.95%	3.04%	83.61%	0.00003%	12.71%	88.45%	3.52%	3.52%
	Amazon0505	91.35%	1.72%	6.08%	0.44%	91.46%	0.11%	3.62%	0.05%
	Amazon0601	0.10%	75.90%	18.74%	0.21%	5.29%	96.02%	2.04%	0.27%
**Social network**	twitter_combined	79.40%	7.23%	12.37%	0.88%	80.04%	0.63%	19.39%	0.34%
	Facebook_0	0.86%	0.58%	86.46%	0.02%	7.49%	89.34%	5.48%	5.48%
	Facebook_107	0.05%	0	54.08%	0.002%	45.92%	99.16%	45.92%	45.92%
	Facebook_348	0.35%	0.17%	38.64%	0.02%	61.19%	99.48%	61.01%	61.01%

Reference:

1. Kwakernaak, H. & Sivan, R.Linear optimal control systems. *Wiley-inter science New York*, 1972.
2. Commault, C., Dion, J. M. & van der Woude, J. W. Characterization of generic properties of linear structured systems for efficient computations. *Kybernetika*.**38(5),**503-520 (2002).
3. Kalman, R. E. Mathematical description of linear dynamical systems. *Journal of the Society for Industrial and Applied Mathematics, Series A: Control*. **1,** 152-192 (1963).
4. Lin, C.T. Structural controllability. *IEEE Transactions on Automatic Control*.**19,** 201-208. (1974).
5. Liu, Y. Y., Slotine, J. J. & Barabasi, A. L. Controllability of complex networks. *Nature*. **473,** 167 (2011).
6. Ruths, J. & Ruths, D. Control profiles of complex networks. *Science*. **343,** 1373 (Mar 21, 2014).
7. Menichetti, G., Dall'Asta, L. & Bianconi, G. Network Controllability Is Determined by the Density of Low In-Degree and Out-Degree Nodes. *Physical Review Letters*. **113,** (2014).
8. Jia, T. & Barabasi, A.L. Emergence of bimodality in controlling complex networks. *Nature communications*. **4,** 2002 (2013).
9. Murota, K. *Matrices and Matroids for Systems Analysis*. (Springer Science & Business Media, 2000)
10. Jia, T. & Barabasi, A. L. Control capacity and a random sampling method in exploring controllability of complex networks. *Sci Rep*. **3,** 2354 (2013).
11. Jia, T. & Posfai, M. Connecting Core Percolation and Controllability of Complex Networks. *Scientific Reports* .**4,** 5379 (2014).
12. Karp, R. M. & Sipser, M. Maximum matchings in sparse random graphs. *Proceedings of the 22th IEEE Symposium on Foundations of Computer Science*. (1981).
13. Lovász, L. & Plummer, M. D. *Matching Theory*. (American Mathematical Soc., vol. 367, 2009).
14. Berge, C. Two theorems in graph theory. *Proceedings of the National Academy of Sciences of the United States*

of America. **43,**842-844 (1957).

15. Gould, R. *Graph Theory, Chapter 7: Matchings and r-Factors*(Benjamin/Cummings Publishing Co., Menlo Park, CA, 1988)
16. Baird, D., Luczkovich, J. & Christian, R. R. Assessment of spatial and temporal variability in ecosystem attributes of the St Marks National Wildlife Refuge, Apalachee Bay, Florida.Estuarine, Coastal, and Shelf. *Science*. **47,** 329-349 (1998).
17. Dunne, J. A., Williams, R. J. & Martinez, N. D. Food-web structure and network theory: the role of connectance and size. *Proc. Natl. Acad. Sci. USA*.**99,** 12917-12922 (2002).
18. Ulanowicz, R. E. & DeAngelis, D. L. Network Analysis of Trophic Dynamics in South Florida Ecosystems. *US Geological Survey Program on the South Florida Ecosystem*. **114,** (2005).
19. Montoya, J. M. & Sol´e, R. V. Small World Patterns in Food Webs. *Journal of Theoretical Biology.* **214,** 405–412(2002).
20. Martinez, N. D. Artifacts or attributes? Effects of resolution on the Little Rock Lake food web. *Ecol. Monogr*. **61,** 367-392 (1991).
21. Watts, D. J. & Strogatz, S. H. Collective dynamics of 'small-world' networks. *Nature* .**393,** 440-442 (1998).
22. Shen-Orr, S. S., Milo, R., Mangan, S. & Alon, U. Network motifs in the transcriptional regulation network of Escherichia coli. *Nature genetics.* **31,** 64 (2002).
23. Balaji, S., Babu, M. M., Iyer, L. M., Luscombe, N. M. & Aravind, L. Comprehensive analysis of combinatorial regulation using the transcriptional regulatory network of yeast. *J. Mol. Biol.* **360,** 213–227 (2006).
24. Milo, R. *et al*. Network motifs: Simple building blocks of complex networks. *Science*. **298,** 824 (2002).
25. Milo, R. *et al*. Superfamilies of evoved and designed networks. *Science*. **303,** 1538-1542 (2004).
26. Van Duijn, M. A. J., Zeggelink, E. P. H., Huisman, M., Stokman, F. N. & Wasseur, F. W. Evolution of sociology freshmen into a friendship network. *J. Math. Sociol*. **27,** 153-191 (2003).
27. Leskovec, J., Lang, K. J., Dasgupta, A. & Mahoney, M. W. Community structure in large networks: Natural cluster sizes and the absence of large well-defined clusters. *Internet Mathematics*. **6,** 29-123(2009).
28. Leskovec, J., Huttenlocher, D. & Kleinberg, J. Predicting positive and negative links in online social networks. *Proceedings of the 19th international conference on World wide web. ACM.*641-650 (2010).
29. Milo, R. *et al*. Network motifs: simple building blocks of complex networks. *Science*. **298,** 824-827 (2002).
30. Leskovec, J., Kleinberg, J. & Faloutsos, C. Graphs over Time: densification laws, shrinking diameters and possible explanations. *ACM SIGKDD International Conference on Knowledge Discovery and Data Mining (KDD)*. 177-187 (2005).
31. de Nooy, W., Mrvar, A. & Batagelj, V. *Exploratory Social Network Analysis with Pajek* (Cambridge Univ. Press, Cambridge, 2004).
32. Handcock, M. S., Hunter, D., Butts, C. T., Goodreau, S. M. & Morris, M. *Statnet: An R package for the Statistical Modeling of Social Networks*. http://www.csde.washington.edu/statnet, (2003).
33. Adamic, L. A. & Glance, N. The Political Blogosphere and the 2004 US Election: divided they blog. *Proceeding LinkKDD '05 Proceedings of the 3rd international workshop on Link discovery*, 36-43 (2005).
34. Albert, R., Jeong, H. & Barabasi, A.-L. Diameter of the world wide web. *Nature*.**401,** 130–131(1999).

35. Ripeanu, M.,Foster, I. &Iamnitchi, A. Mapping the Gnutella Network: Properties of Large-Scale Peer-to-Peer Systems and Implications for System Design. *IEEE Internet Computing Journal*(2002).
36. Opsahl, T. & Panzarasa, P. Social entworks Clustering in weighted networks. *Social. Network*. **31,** 155-163 (2009).
37. Leskovec, J., Adamic L. & Adamic, B. The Dynamics of Viral Marketing. *ACM Transactions on the Web (ACM TWEB)*, **1,** Issue 1 (2007).
38. McAuley, J. & Leskovec, J. Learning to Discover Social Circles in Ego Networks. *NIPS*.**2012,** 548-556 (2012).
